# Multitask learning for biomedical named entity recognition with cross-sharing structure

**DOI:** 10.1186/s12859-019-3000-5

**Published:** 2019-08-16

**Authors:** Xi Wang, Jiagao Lyu, Li Dong, Ke Xu

**Affiliations:** State Key Laboratory of Software Development Environment, Beihang University, Beijing, 100191 China

**Keywords:** Multi-task learning, Named entity recognition, Cross-sharing structure

## Abstract

**Background:**

Biomedical named entity recognition (BioNER) is a fundamental and essential task for biomedical literature mining, which affects the performance of downstream tasks. Most BioNER models rely on domain-specific features or hand-crafted rules, but extracting features from massive data requires much time and human efforts. To solve this, neural network models are used to automatically learn features. Recently, multi-task learning has been applied successfully to neural network models of biomedical literature mining. For BioNER models, using multi-task learning makes use of features from multiple datasets and improves the performance of models.

**Results:**

In experiments, we compared our proposed model with other multi-task models and found our model outperformed the others on datasets of gene, protein, disease categories. We also tested the performance of different dataset pairs to find out the best partners of datasets. Besides, we explored and analyzed the influence of different entity types by using sub-datasets. When dataset size was reduced, our model still produced positive results.

**Conclusion:**

We propose a novel multi-task model for BioNER with the cross-sharing structure to improve the performance of multi-task models. The cross-sharing structure in our model makes use of features from both datasets in the training procedure. Detailed analysis about best partners of datasets and influence between entity categories can provide guidance of choosing proper dataset pairs for multi-task training. Our implementation is available at https://github.com/JogleLew/bioner-cross-sharing.

## Background

Biomedical named entity recognition (BioNER) aims at annotating named entity mentions with their entity types (e.g., genes, proteins [1], and diseases [2]) in the input biomedical text. The outputs of model indicate not only the locations of entity mentions but also their types. BioNER models provide useful information for downstream tasks of biomedical literature mining, such as entity relation extraction [3–5], and biomedical network construction [6–8].

BioNER task requires to detect boundaries of biomedical entities and predict their entity types. Most previous systems treat the task as a sequence labeling problem. Traditional neural network models for BioNER rely on features designed for each task. These BioNER models use hand-crafted rules [9] and domain-specific features [10], such as orthographic features, morphological features [11–14]. The drawback of these neural network models is that features are specially designed for each dataset or each entity type in order to achieve good performance; thus, features used in one BioNER model may not work well in another. Recent studies showed that the neural network model is capable of feature generation work without manual choosing. Some of these models use bi-directional Long Short-Term Memory with Conditional Random Field (BiLSTM-CRF) [15], and other models have extra character-level CNN [16, 17] or character-level LSTM [18, 19] to capture character features of entities.

Recently, multi-task learning (MTL) [20] has been adopted successfully to applications of biomedical literature mining, such as drug discovery [21], entity linking [22]. The multi-task model trains several datasets at the same time, and transfers domain information between datasets. By sharing representations between the main task and auxiliary task, the multi-task model improves the performance on the main task. For MTL BioNER models, the number of successful examples is growing. Crichton et al. [23] uses convolution layer as the shared part and fully connected layer as task-specific part. Wang et al. [19] experiments shared character Bi-LSTM, shared word Bi-LSTM, and shared both. Although the multi-task model can optimize the performance of the main dataset, using different combinations of training datasets may have discrepancy performances. Some other models use special methods to improve performance, such as adversarial loss [24], label-aware MMD [25], Learn What to Share Structure [26].

In this paper, we compare some different multi-task models and propose our new model with the cross-sharing structure for BioNER. No hand-crafted feature is required in our model. The proposed model is based on the BiLSTM-CNN-CRF model [16] which is a single-task neural network model. In our model, shared Bi-LSTM unit is used to learn the shared features, and private Bi-LSTM units are for the task-specific features. Besides, a cross-sharing structure helps to share information between private units. We compare the proposed model with other multi-task models [19, 24] on four main datasets of different domains. We also discover the influence of dataset pairs and dataset size to the performance of our proposed model. Results demonstrate that the proposed model achieves good results. Our method provides a novel structure of multi-task sharing in BioNER task and improves the overall performance on BioNER datasets.

## Preliminaries

In this section, some basic concepts related to our multi-task neural network are introduced.

### Bi-directional long short-Term memory (Bi-LSTM)

Long Short-Term Memory (LSTM) [27] is a special edition of Recurrent neural network (RNN), and LSTM avoids the gradient vanishing or exploding problems appearing in RNN. A normal LSTM cell contains a input gate, a output gate and a forget gate, and there are connections between these gates. We denote ***X***={***x***_1_,***x***_2_,...,***x***_*T*_} as the series input of LSTM, where *T* is the sequence length of input vector. The output of LSTM is a sequence of vector ***H***={***h***_1_,***h***_2_,...,***h***_*T*_}. The LSTM cell calculates ***h***_*t*_ via the following calculation:

1 $$\begin{array}{*{20}l} \boldsymbol{f}_{t} &= \sigma (\boldsymbol{W}_{f} [\boldsymbol{h}_{t-1}, \boldsymbol{x}_{t}] + \boldsymbol{b}_{f}) \end{array} $$

2 $$\begin{array}{*{20}l} \boldsymbol{i}_{t} &= \sigma (\boldsymbol{W}_{i} [\boldsymbol{h}_{t-1}, \boldsymbol{x}_{t}] + \boldsymbol{b}_{i}) \end{array} $$

3 $$\begin{array}{*{20}l} \tilde{\boldsymbol{C}_{t}} &= tanh (\boldsymbol{W}_{C} [\boldsymbol{h}_{t-1}, \boldsymbol{x}_{t}] + \boldsymbol{b}_{C}) \end{array} $$

4 $$\begin{array}{*{20}l} \boldsymbol{C}_{t} &= \boldsymbol{f}_{t} \odot \boldsymbol{C}_{t-1} + \boldsymbol{i}_{t} \odot \tilde{\boldsymbol{C}_{t}} \end{array} $$

5 $$\begin{array}{*{20}l} \boldsymbol{o}_{t} &= \sigma (\boldsymbol{W}_{o} [\boldsymbol{h}_{t-1}, \boldsymbol{x}_{t}] + \boldsymbol{b}_{o}) \end{array} $$

6 $$\begin{array}{*{20}l} \boldsymbol{h}_{t} &= \boldsymbol{o}_{t} \odot tanh (\boldsymbol{C}_{t}) \end{array} $$

In these equations, ⊙ denotes element-wise multiplication. *σ* and *tanh* are element-wise sigmoid function and tanh function, respectively. ***f***_*t*_,***i***_*t*_,***o***_*t*_ are the forget gate, the input gate, and the output gate, respectively. $ \tilde {\boldsymbol {C}_{t}} $ indicates some information from current input applied to cell state. ***h***_*t*_ calculates the cell output by the input and current cell state. ***W***_*j*_,***b***_*j*_(*j*=*f*,*i*,*C*,*o*) are the trainable parameters. The LSTM cell is designed to avoid the long-term dependency problem, and it is capable of capturing information for long periods.

**Fig. 1:**
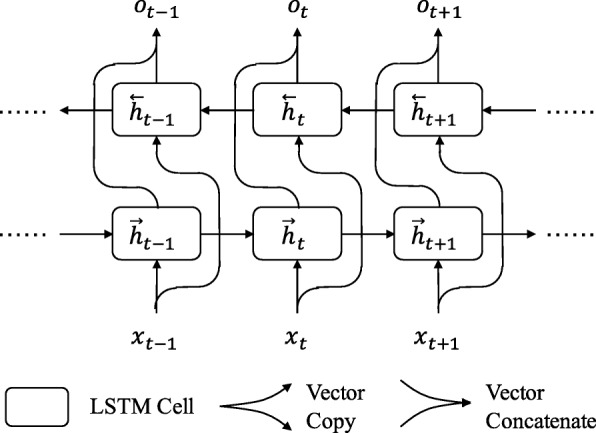
Bi-LSTM Structure. The figure displays a part of Bi-LSTM network. Input vectors are fed to two directions of LSTM, and the output of two directions of LSTM is concatenated as the whole output

Bi-LSTM is the two-direction version of LSTM. For original LSTM, the cells take input in one direction, so ***h***_*t*_ will capture some information only from previous LSTM cells. In order to capture the information from the following cells, another set of LSTM cells is used in Bi-LSTM. As shown in Figure 1, the bi-directional long short-term memory (Bi-LSTM) model contains two directions of LSTM network, original direction and reversed direction.

7 $$ \overrightarrow{\textbf{h}}_{t} = LSTM\left(\overrightarrow{\textbf{h}}_{t - 1}, {\textbf{x}}_{t}\right)  $$

8 $$ \overleftarrow{\textbf{h}}_{t} = LSTM\left(\overleftarrow{\textbf{h}}_{t + 1}, {\textbf{x}}_{t}\right)  $$

9 $$ \textbf{o}_{t} = \overrightarrow{\textbf{h}}_{t} \oplus \overleftarrow{\textbf{h}}_{t}  $$

In these equations, $ \overrightarrow {\boldsymbol {h}}_{t} $ and $ \overleftarrow {\boldsymbol {h}}_{t} $ are the cells output of two directions. ⊕ denotes vector concatenation. The vectors, $ \overrightarrow {\boldsymbol {h}}_{t} $ and $ \overleftarrow {\boldsymbol {h}}_{t} $, are concatenated as the final output. In this way, ***o***_*t*_ keeps the information from previous and following LSTM cells.

### Conditional random field (CRF)

Conditional Random Field (CRF) [28] is a conditional probability distribution model and widely used in sequence labeling tasks to generate new tag based on recent tags. When a set of random variables are given as input, CRF outputs another set of random variables according to some rules. For example, in biomedical NER task with IOB annotation, the tag after B-Gene can be I-Gene rather than I-Disease. If the previous tag is B-Gene, CRF would output I-Disease in a low probability to avoid the error of mixing different types of tags. CRF has been adopted in many state-of-art models to help to generate meaningful and legal annotations.

Let the input of CRF is vector ***Z***=(***z***_1_,***z***_2_,...,***z***_*n*_), and the generated output sequence is $ \boldsymbol {\hat {Y}} = (\hat {y}_{1}, \hat {y}_{2},..., \hat {y}_{n}) $. For BioNER task, the input ***z***_*i*_ can be a feature vector representing the *i*th word. CRF model describes the probability of generating the whole label sequence based on ***Z***, shown as below:

10 $$ p(\boldsymbol{\hat{Y}}|\boldsymbol{Z}; \boldsymbol{W}, \boldsymbol{b})=\frac{\prod_{i=1}^{n} f_{i}(\hat{y}_{i-1}, \hat{y}_{i}, \boldsymbol{Z})}{\sum_{y' \in\phi(\boldsymbol{Z})} \prod_{i=1}^{n} f_{i}(y'_{i-1}, y'_{i}, \boldsymbol{Z})}  $$

In this equation, *ϕ*(***Z***) represents all of the possible label sequences for *Z*. The function $ f_{i}(y_{j}, y_{k}, \boldsymbol {Z}) = exp(\boldsymbol {W}_{y_{j},y_{k}}\boldsymbol {z_{i}} + \boldsymbol {b}_{y_{j},y_{k}}) \phantom {\dot {i}\!}$, where the weight $\phantom {\dot {i}\!} \boldsymbol {W}_{y_{j},y_{k}} $ and the bias $ \boldsymbol {b}_{y_{j},y_{k}} \phantom {\dot {i}\!}$ are the trainable parameters corresponding to the pair of labels (*y*_*j*_,*y*_*k*_).

In the training procedure, we use the negative log-likelihood function to calculate the loss function *J* and find the optimal sequence *y*^∗^ by minimum the loss function. The Viterbi algorithm is used to calculate the loss and the optimal sequence.

11 $$\begin{array}{*{20}l} J(\boldsymbol{W}, \boldsymbol{b}) &= -\sum_{i} \log(p(\boldsymbol{\hat{Y}}|\boldsymbol{Z}; \boldsymbol{W}, \boldsymbol{b})) \end{array} $$

12 $$\begin{array}{*{20}l} y^{*} &= \underset{y \in \phi(\boldsymbol{Z})}{\arg\min} \ \ J(\boldsymbol{W}, \boldsymbol{b}) \end{array} $$

## Methods

In this section, we introduce our baseline single-task model and some multi-task models for BioNER tasks.

### Baseline single-task model (STM)

**Fig. 2:**
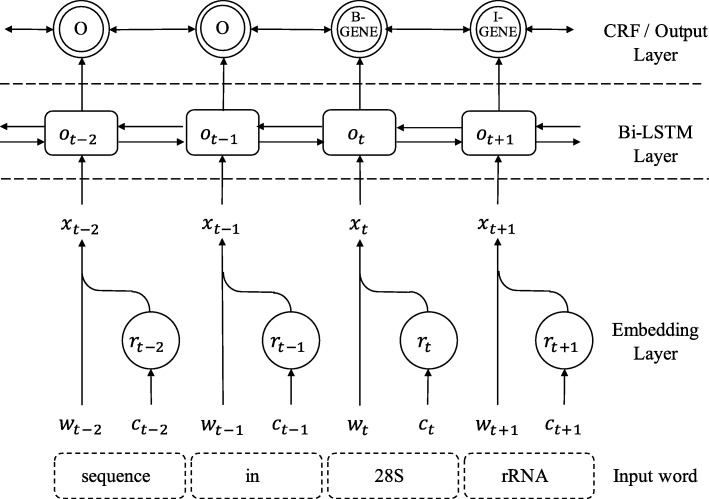
Single-task Model (STM). The input is a sentence from the BioNER dataset. The dotted rectangles represent words in a sentence, and the solid rectangles represent Bi-LSTM cells. The circles represent CNN units, and the double circles represent CRF units. The tags in the double circles, e.g., “O”, “B-GENE”, are the output of the CRF layer

We choose the model from Ma and Hovy [16] as our baseline single-task model. Unlike the vanilla BiLSTM-CRF model, this model uses an extra CNN layer to capture character-level features. All the multi-task models in the paper are implemented based on this single-task model; thus, we choose it as our baseline model. The model structure is shown in Fig. 2.

For simplicity, ***w***_*t*_ denotes word embedding of word *t* and the ***c***_*t*_ denotes character embeddings of word *t*. The shape of ***c***_*t*_ is *d*_*c*_ by *l*_*c*_, where *d*_*c*_ is the dimension of character embedding and *l*_*c*_ is the count of characters in the word.

In the embedding layer, the character representation ***r***_*t*_ is calculated based on character embedding ***c***_*t*_ by CNN to extract morphological information. The CNN scheme we use is the same as Ma and Hovy [16]. The convolution has the filter size of *d*_*c*_ by *l*_*f*_ and padding length of *l*_*f*_−1, where *l*_*f*_ is a hyperparameter. After the convolution calculation, the output is a new vector of shape *d*_*c*_ by (*l*_*c*_+*l*_*f*_−1). Then max pooling is used to produce a vector of size *d*_*c*_ as the final char representation ***r***_*t*_. A dropout layer is adopted at the input of CNN. Finally, word embedding ***w***_*t*_ and character representation ***r***_*t*_ are concatenated as ***x***_*t*_.

After the embedding layer, resulting sequence of embeddings ***X***={***x***_1_,***x***_2_,...,***x***_*n*_} are fed into Bi-LSTM layer to get ***O***={***o***_1_,***o***_2_,...,***o***_*n*_}. Two dropout layers are applied at the input and output of the Bi-LSTM layer. The Bi-LSTM layer is used to extract information from the word representation ***x***_*t*_.

The top layer of the model is the CRF layer. This layer takes output vectors ***O*** to predict label sequences. As shown in Fig. 2, the word “28S” and the word “rRNA” are predicted as B-Gene and I-Gene, respectively, which suggests that the model recognizes the entity “28S rRNA”.

### Fully-shared multi-task model (FS-MTM)

Our fully-shared multi-task model is based on MTM-CW from Crichton et al. [23]. All the multi-task models in this paper are designed for two datasets. If modifications applied, these models are suitable for three or more datasets. The embedding layer, Bi-LSTM layer and CRF layer in the multi-task models are the same as those in the baseline single-task model.

**Fig. 3:**
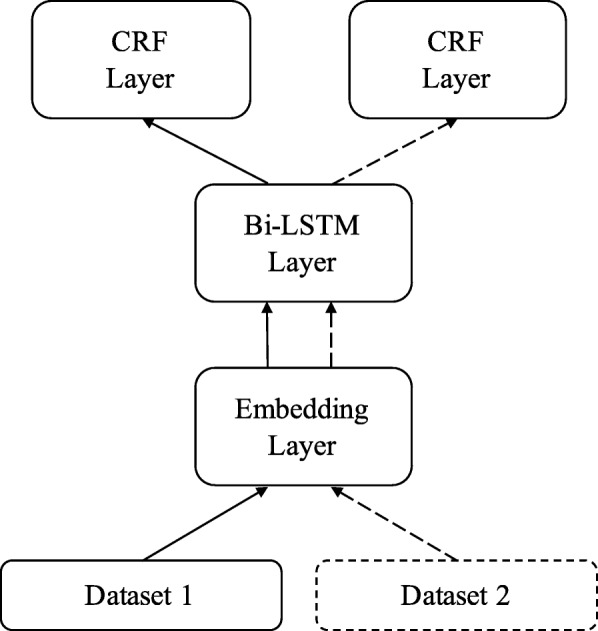
Fully-shared Multi-task Model (FS-MTM). The embedding layer and the Bi-LSTM layer are shared by two datasets, and two CRF layer are used for two datasets

In the fully-shared multi-task model, we use an embedding layer and a Bi-LSTM layer as shared parts, and two CRF layers for two datasets, as shown in Fig. 3. When training and testing, word embeddings and character embeddings are first fed to the embedding layer, and then the Bi-LSTM layer takes the output of embedding layer. In the end, the output of Bi-LSTM is fed to one of the CRF layers. If source data is from dataset 1, CRF layer for dataset 1 is activated with another CRF layer ignored, and vice versa. In this model, Bi-LSTM captures all the features of dataset 1 and 2, and CRF layer produces different tags according to the input dataset.

### Shared-private multi-task model (SP-MTM)

**Fig. 4:**
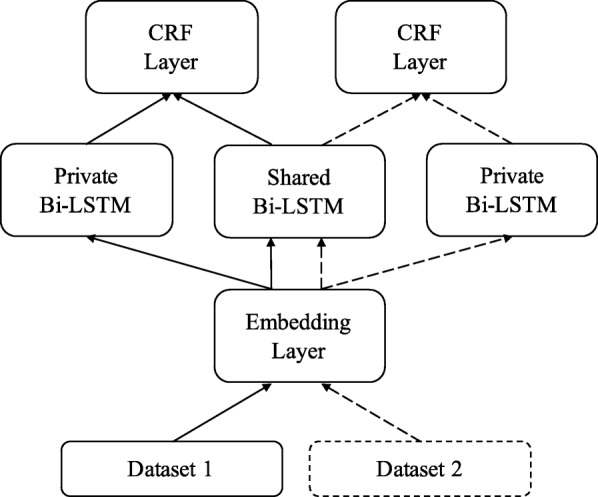
Shared-private Multi-task Model (SP-MTM). The embedding layer and shared Bi-LSTM are shared by two datasets. Two CRF layer and two private Bi-LSTMs are used for two datasets

Our shared-private multi-task model is based on SP-MTL from Liu et al. [24]. As shown in Fig. 4, there are two private Bi-LSTMs for two tasks and one shared Bi-LSTM. Word embeddings and character embeddings are first fed to the embedding layer. Then the output of the embedding layer is replicated and fed into shared Bi-LSTM and corresponding private Bi-LSTM, according to the source dataset. Finally, the output of shared and private Bi-LSTMs are concatenated and fed into corresponding CRF layer. In this model, shared Bi-LSTM and private Bi-LSTM captures shared and task-independent features, respectively. CRF layer produces different tags based on task-related feature representations.

### Adversarial multi-task model (ADV-MTM)

**Fig. 5:**
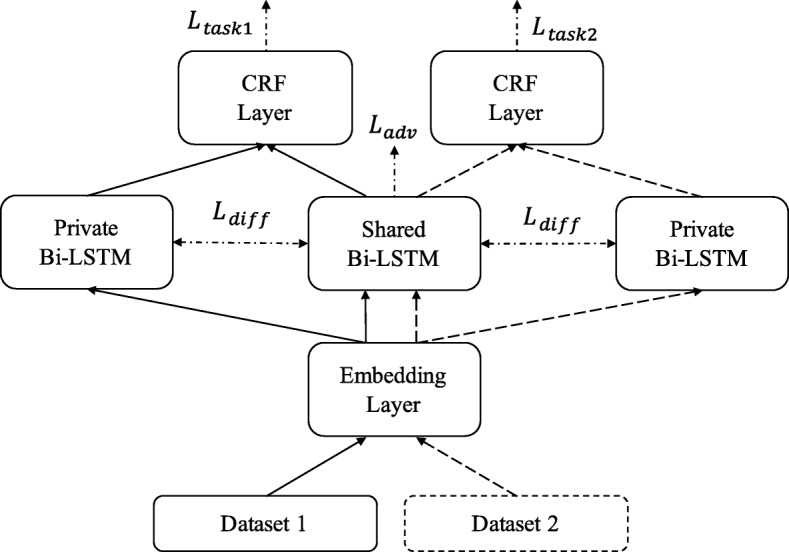
Adversarial Multi-task Model (ADV-MTM). The embedding layer and shared Bi-LSTM are shared by two datasets. Two CRF layer and two private Bi-LSTMs are used for two datasets. Three kinds of losses are marked on the figure

As shown in Fig. 5, our adversarial multi-task model is based on the adversarial shared-private model from Liu et al. [24]. The basic network structure of the adversarial multi-task model is the same as the shared-private multi-task model, but the calculation of loss is different.

We deem the current data as *d*, and source datasets are $ \mathcal {D}_{1}, \mathcal {D}_{2} $. *L*_*task*_ is the task loss calculated by CRF layer. shown as Eq. 13.

13 $$  L_{task} = \left\{ \begin{array}{lr} L_{task1}, & d \in \mathcal{D}_{1}; \\ L_{task2}, & d \in \mathcal{D}_{2}. \end{array} \right.  $$

*L*_*diff*_ is calculated by the output of shared Bi-LSTM and private Bi-LSTM. *L*_*diff*_ describes the similarity of these two output vectors. Minimizing *L*_*diff*_ encourages shared and private Bi-LSTM to extract different features of input. *L*_*diff*_ is calculated as Eq. 14:

14 $$  L_{diff} = \sum_{k=1, 2}||{\boldsymbol{S}}^{\top}\boldsymbol{P}^{k}||_{F}^{2}  $$

where ***S*** is the output of shared Bi-LSTM and ***P***^*k*^ is the output of private Bi-LSTM of dataset *k*. $ ||\cdot ||_{F}^{2} $ is the squared Frobenius norm.

*L*_*adv*_ is task adversarial loss. The shared Bi-LSTM can be regarded as generative model G which produce vector to hide the information of source dataset, and we use a discriminative model D to identify the source dataset against generative model G. Discriminative model D is shown as Eq. 15:

15 $$  D(\boldsymbol{s}_{T}^{k}, \theta_{D}) = softmax\left(\boldsymbol{W}\boldsymbol{s}_{T}^{k} + \boldsymbol{b}\right)  $$

where $ \boldsymbol {s}_{T}^{k} $ is the output of shared Bi-LSTM of dataset *k* at time *T*. ***W*** and ***b*** are trainable parameters. And the adversarial loss function is:

16 $$  L_{adv} = -\max_{\theta_{G}}\left(\min_{\theta_{D}}\left(\sum_{k=1}^{K} \boldsymbol{d}_{i}^{k} \log\left[D\left(E\left(\boldsymbol{x}^{k}\right)\right)\right]\right)\right)  $$

Discriminative model D is able to recognize source dataset by task-dependent features, and generative model G tends to keep common features to confuse discriminative model D; therefore, minimizing *L*_*adv*_ encourages shared Bi-LSTM to keep more shared features of two datasets.

The final loss is the weighted sum of these three kinds of losses.

17 $$ L = L_{task} + \alpha L_{adv} + \beta L_{diff}  $$

where *α* and *β* are hyperparameters.

Grid search can be used to find the optimized hyperparameters *α* and *β*. By using the gradient reversal layer [29] before the discriminative model, the whole network can be trained with backpropagation.

### Multi-task model with cross-sharing structure (CS-MTM)

In this section, we introduce our multi-task model with cross-sharing structure. This model captures features from both datasets and takes advantage of all the feature representations.

**Fig. 6:**
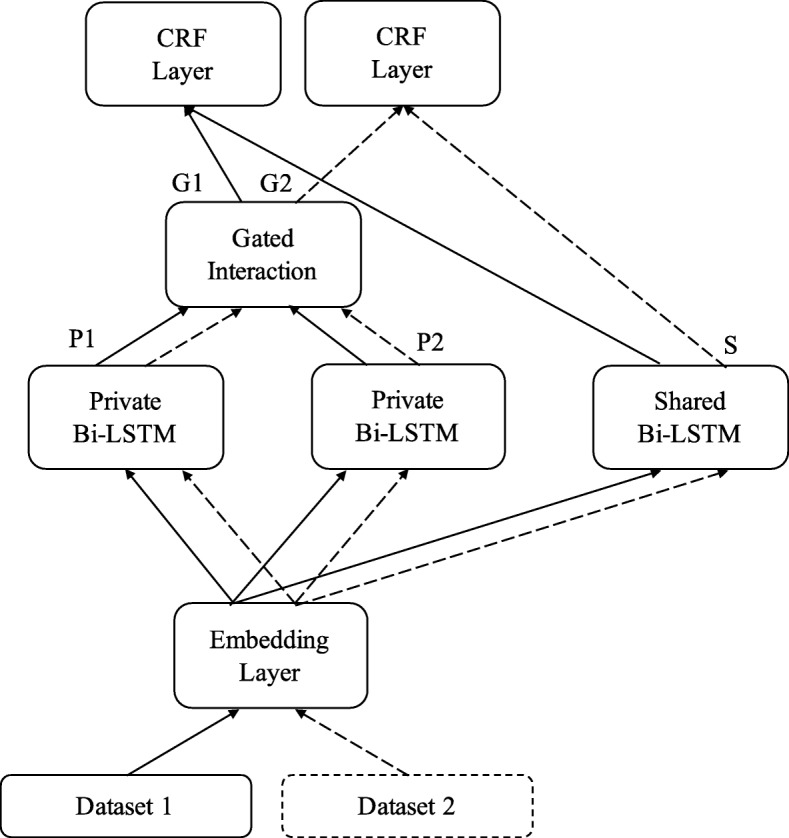
Cross-sharing Multi-task Model (CS-MTM). The embedding layer and shared Bi-LSTM are shared by two datasets. Gated interaction unit is used to adjust the output of private Bi-LSTMs. *P*_1_,*P*_2_: Output of private Bi-LSTMs. *S*: Output of the shared Bi-LSTM. *G*_1_,*G*_2_: Output of the gated interaction unit

As shown in Fig. 6, the word embeddings and character embeddings of the input sentence are first fed to the embedding layer. The structure of the embedding layer is the same as that in the baseline single-task model. The embedding layer captures the information in word embeddings and character embeddings. The output of the embedding layer is the word representations, which can be used in the Bi-LSTM layers.

After the embedding layer, the word representations are replicated as the input of shared Bi-LSTM and **both** private Bi-LSTMs. ***P***_1_,***P***_2_ denote the output of two private Bi-LSTMs. ***S*** denotes the output of shared Bi-LSTM. Intuitively, the private Bi-LSTMs are used to capture task-independent features; thus, ***P***_1_,***P***_2_ are the feature representations of dataset 1 and 2. The shared Bi-LSTM captures the common features from both datasets and ***S*** is the representation of common features.

In our previous SP-MTM and ADV-MTM, either ***P***_1_ or ***P***_2_ is calculated depending on source dataset. In this way, only feature representation of source dataset is calculated, but the other feature representation which may still be useful is not calculated. In multi-task learning, using information from other datasets to improve the performance of origin dataset is the main idea, so both ***P***_1_ and ***P***_2_ are used in this model.

The gated interaction unit then takes ***P***_1_,***P***_2_ as input and produces a mixed feature representation. ***G***_1_,***G***_2_ denote the output of gated interaction unit for two datasets. Eq. 18 and (19) show how gated interaction unit works.

18 $$\begin{array}{*{20}l}  \boldsymbol{G}_{1} &= \boldsymbol{P}_{1} \odot \sigma(\boldsymbol{W}_{2 \rightarrow 1}\boldsymbol{P}_{2} + \boldsymbol{b}_{2 \rightarrow 1}) \end{array} $$

19 $$\begin{array}{*{20}l} \boldsymbol{G}_{2} &= \boldsymbol{P}_{2} \odot \sigma(\boldsymbol{W}_{1 \rightarrow 2}\boldsymbol{P}_{1} + \boldsymbol{b}_{1 \rightarrow 2}) \end{array} $$

where ⊙ is element-wise multiplication, *σ* is a sigmoidal function, and ***W***_1→2_,***W***_2→1_,***b***_1→2_,***b***_2→1_ are trainable parameters.

We deem the current data as *d*, and source datasets are $ \mathcal {D}_{1}, \mathcal {D}_{2} $. The final output of gated interaction unit *G* is determined by the source dataset, shown as Eq. 20.

20 $$  \boldsymbol{G}=\left\{ \begin{array}{lr} \boldsymbol{G}_{1}, & d \in \mathcal{D}_{1}; \\ \boldsymbol{G}_{2}, & d \in \mathcal{D}_{2}. \end{array} \right.  $$

In the gated interaction unit, two private feature representations ***P***_1_,***P***_2_ share feature information with each other. When training, four trainable parameters are adjusting to learning what to share between two representations. For dataset 1, ***P***_2_ contains the information of features from dataset 2, and these features are task-independent and cannot be used directly to improve the performance of dataset 1; otherwise, these features should be captured by shared Bi-LSTM. The operation in gated interaction unit provides an indirect way to make use of the information in ***P***_2_. In this way, both feature representations ***P***_1_,***P***_2_ are used to produce a new mixed feature representation.

Before the CRF layer, output vectors of gated interaction unit and shared Bi-LSTM are concatenated, shown as Eq. 21.

21 $$  \boldsymbol{V} = \boldsymbol{G} \oplus \boldsymbol{S}  $$

In this way, information of shared feature representation and private feature representation is combined and fed to the CRF layer. CRF layer produces predicted tags based on ***V***.

## Experiment settings

In this section, we introduce our datasets, evaluation metrics, and training details.

### Datasets

We conduct experiments on several BioNER datasets from Crichton et al. [23]. The detailed information about the datasets used in our experiments is listed in Table 1. We use datasets with IOB format. These datasets are available to the open, and you can access https://github.com/cambridgeltl/MTL-Bioinformatics-2016 to get these datasets.

**Table 1:** Biomedical NER datasets used in the experiments

Dataset	Size	Entity types & counts
BC2GM	20,131 sentences	Gene (24,583)
Ex-PTM	3,653 sentences	Protein (4,698)
NCBI-disease	7,287 sentences	Disease (6,881)
Linnaeus	23,155 sentences	Species (4,263)
JNLPBA	24,806 sentences	Cell (12,969), Gene (10,589), Protein (35,336)
BC5CDR	13,938 sentences	Chemical (15,935), Disease (12,852)
BioNLP09	11,356 sentences	Protein (14,963)
BioNLP11ID	5,178 sentences	Chemical (973), Protein (6,551), Species (3,471)
BioNLP13PC	5,051 sentences	Cell (1,013), Chemical (3,989), Gene (10,891)

As these datasets use various BioNER tags to mark out entities, we divide them into six categories: Cell, Chemical, Disease, Gene, Protein and Species. For the entity types column in Table 1, BioNER tags are counted according to these six categories. In different datasets, BioNER tags belonging to the same category may vary. For example, in Gene categories, B-GENE/I-GENE tags are used in BC2GM dataset, while B-DNA/I-DNA are in JNLPBA dataset. In our experiments, tags are kept as they are rather than changed to be the same.

In our multi-task models, two datasets are used in the training procedure. We focus on one dataset and try to optimize the performance on it. This dataset is called the main dataset, and the other is called the auxiliary dataset. By observation, we find that some datasets contain entities from just one category, while some others from multiple categories. In order to diminish the influence between different entity categories, we prefer datasets which contain entities from one category to be main datasets. In our experiments, BC2GM, Ex-PTM, NCBI-disease, and Linnaeus are chosen as main datasets, and auxiliary datasets are picked from JNLPBA, BioNLP09, BioNLP11ID, BioNLP13PC, and BC5CDR. The performance of the main datasets is recorded in experimental results.

### Evaluation metrics

We use the training set and the development set to train the model, and report the performance on the test set. We deem each predicted tag is correct only if it is the same as the ground-truth tag. We calculate macro-averaged precision, recall, F1 scores of main dataset, and these scores are recorded as final dataset scores.

### Training details

**Word embeddings** We use pre-trained word vectors of GloVe model, and the pre-trained corpus is Wikipedia 2014 + Gigaword 5 (6B tokens, 400K vocab). The dimension of word vectors is 100.

**Character embeddings** The dimension of character embeddings *d*_*c*_ is 30. Number of filters in CNN is 30. *l*_*f*_ in the CNN is set to 3.

**Bi-LSTM layers** Bi-LSTM in our model uses the same hyperparameters, including Bi-LSTM in baseline single-task model, and shared/private Bi-LSTM in multi-task models. We set the dimension of hidden vectors to 256. For Bi-LSTM layers in all of our models, we use a linear unit to reshape hidden vectors to 128-dimensional vector as output. The dropout rate of all the dropout layers is 0.5.

**CRF layers** We use Linear-chain CRF to train and test. The Viterbi algorithm is used in the training procedure.

**Training settings** Our training procedure contains 80 epochs. Parameter optimization is performed with RMSprop. The decay rate of RMSProp is set to 0.95, and momentum is set to 0. Batch size is set to 16. Learning rate is 0.001 at initial, and decay at the end of every epoch at the rate of 3%. Besides, We use gradient clipping to limit max L2 norm of the gradients to 5.0 in order to avoid gradient exploding.

**MTM training** When performing multi-task training, batches of data from 2 datasets train in turns. To be specific, a batch of data from dataset 1 is used to train, then a batch of data from dataset 2 is used to train, this procedure is regarded as a turn. In one turn, two batches of data are randomly picked from their source datasets. In an epoch, the number of turns is set to the number of batches in the main dataset. In this case, we ensure the main dataset to be fully trained.

**Grid search** For the adversarial multi-task model, grid search is used to find the hyperparameters *α* and *β*. We try out *α* from {0, 0.1, 0.01}, and *β* from {0, 0.01, 0.001}. An extra gradient reverse layer is applied before the task discriminator unit in order to train the model with gradient descent.

## Results

In this section, we display and analyze the experiment results, and compare our proposed model with related ones.

### Performance comparison

We compare the baseline single-task model (STM) and other multi-task models (MTM). The results are shown in Table 2. It shows the performance (precision, recall, F1) of different models on four target datasets. The bold number in one row indicates the best F1 score for the dataset.

**Table 2:** Model Performance Comparison

		Baseline Single-task Model (STM)	Fully-shared Multi-task Model (FS-MTM)	Shared-private Multi-task Model (SP-MTM)	Adversarial Multi-task Model (ADV-MTM)	Cross-sharing Multi-task Model (CS-MTM)
BC2GM	Precision	84.00	83.34	84.51	83.66	83.12
	Recall	83.82	84.75	84.17	84.05	85.74
	F1	83.91	84.04	84.34	83.85	**84.41**
Ex-PTM	Precision	70.83	72.56	70.45	76.60	74.73
	Recall	64.12	70.46	70.03	67.43	69.56
	F1	67.31	71.49	70.24	71.72	**72.05**
NCBI-disease	Precision	88.45	84.39	87.11	86.02	86.59
	Recall	83.78	86.61	85.49	86.86	86.42
	F1	86.05	85.49	86.29	86.44	**86.50**
Linnaeus	Precision	92.86	92.66	93.00	93.74	89.81
	Recall	67.62	66.76	73.86	73.81	76.12
	F1	78.25	77.60	82.33	**82.59**	82.40

Bold: the best F1 score for the dataset

FS-MTM achieves better performance than STM on BC2GM and Ex-PTM datasets but degrades on other two datasets. FS-MTM uses the most basic multi-task structure, and the only shared Bi-LSTM may not separate task-specific features for each task.

SP-MTM improves the performance comparing to FS-MTM and STM, also achieves higher F1 score than baseline STM on all of four main datasets. Intuitively, the private Bi-LSTMs are added and capable of capturing task-specific features.

We observe that both the ADV-MTM and CS-MTM improve the performance of STM, and especially CS-MTM achieves higher F1 score than baseline STM on all of four datasets. On BC2GM dataset, improvements of ADV-MTM are marginal compared with STM. Besides, CS-MTM outperforms ADV-MTM in F1 score on BC2GM, Ex-PTM, and NCBI-disease datasets. Comparing the structure of ADV-MTM and CS-MTM to SP-MTM, it indicates that the adversarial loss calculation and cross-sharing structure could help to improve the performance.

According to the precision and recall score of datasets, CS-MTM tends to produce a higher recall score, and ADV-MTM tends to improve the precision score. Intuitively, minimizing the adversarial loss in ADV-MTM helps to separate shared features and task-specific features and reduce the number of false positives. Unlike ADV-MTM, gated interaction unit in CS-MTM makes use of both feature representations, resulting in less number of false negatives.

When training, we find that the performance of ADV-MTM is not very stable, and the adversarial model uses more epochs to converge. This model has limited performance improvement comparing to SP-MTM and exposes the weakness of GAN.

We list the trainable parameter number of each model in Table 3. In the table, the parameter numbers of STM and FS-MTM are close, and SP-MTM, ADV-MTM, CS-MTM have more parameters. We can conclude that the gated interaction unit in CS-MTM has only a few parameters but improves the overall performance. It suggests that our performance improvement is not just based on the increase in the huge amount of parameters.

**Table 3:** Parameter numbers of all models

Model	Number
STM	3.68M
FS-MTM	3.68M
SP-MTM	5.41M
ADV-MTM	5.41M
CS-MTM	5.44M

### Performance with different auxiliary datasets

Different dataset pairs could produce different results in multi-task learning. We try out all the combinations of one main dataset and one auxiliary dataset. The results are shown in Table 4. The numbers in the table are the F1 scores of dataset pairs. BC2GM, Ex-PTM, NCBI-disease, and Linnaeus are the main dataset that we focus on. The bold number in one row indicates the best F1 score for the dataset. The *↑* / *↓* indicates the positive/negative improvement comparing to STM.

**Table 4:** Performance with different auxiliary datasets

	JNLPBA	BC5CDR	BioNLP 09	BioNLP 11ID	BioNLP 13PC
BC2GM	**84.41** *↑*	84.11 *↑*	83.85	84.15 *↑*	83.90
Ex-PTM	68.81 *↑*	67.51 *↑*	**72.05** *↑*	68.89 *↑*	70.87 *↑*
NCBI-disease	86.17 *↑*	85.74 *↓*	**86.50** *↑*	84.90 *↓*	85.63 *↓*
Linnaeus	78.07 *↓*	**82.40** *↑*	81.93 *↑*	78.46 *↑*	78.37 *↓*

Bold: the best F1 score for the dataset. *↑* / *↓*: positive / negative improvement comparing to STM

From experiment results, JNLPBA is the best partner for BC2GM, and BC5CDR, BioNLP09 are helpful to BC2GM. All these five auxiliary datasets are helpful to Ex-PTM, but the best partner of Ex-PTM is BioNLP09. As for NCBI-disease, BioNLP09 is the best partner, and JNLPBA is also helpful. Auxiliary datasets except JNLPBA and BioNLP13PC are helpful to Linnaeus, and BC5CDR improves its performance significantly.

In auxiliary datasets, JNLPBA is of the biggest size, and BioNLP13PC is the smallest. Using JNLPBA as the auxiliary dataset still degrades on Linnaeus dataset, while using BioNLP13PC as the auxiliary dataset in this experiment improves the performance on Ex-PTM. For these five auxiliary datasets, we cannot observe a tendency of performance increasing or decreasing with the size of dataset changing. This phenomenon indicates that the size of the dataset is not the major factor of performance. If auxiliary dataset lacks beneficial information for the main dataset, the performance of multi-task model would be unfavorable.

BC2GM contains gene tags, and its best partner JNLPBA also contains gene tags. The situation is similar for Ex-PTM and BioNLP09. It could indicate that the dataset pair could work if the auxiliary dataset contains the categories of tags that main dataset also has. But for Linnaeus and its best partner BC5CDR, although they share no same categories of tags, BC5CDR can still provide biomedical information of other categories which is helpful to Linnaeus.

In conclusion, there is no simple rule to find the best partner, the most accurate way is to try out all the combinations.

### Performance with different entity types in auxiliary datasets

In our five auxiliary datasets, some of them contain multiple categories of tags. In order to discover which category of tags is the major factor of performance, we use sub-datasets to perform the experiments. The BC5CDR, BioNLP11ID, BioNLP13PC datasets provide sub-datasets that contain the single category of tags. In this experiments, We choose our four main datasets and BioNLP11ID-chem (Chemical), BioNLP11ID-ggp (Protein), BioNLP11ID-species (Species) as auxiliary datasets. This experiment aims to check which category of tags is the most important for main datasets in CS-MTM. The results are shown in Table 5. The *↑* / *↓* indicates the positive/negative improvement comparing to STM.

**Table 5:** Performance with different entity types in BioNLP11ID

	BioNLP11 ID	BioNLP11 ID-chem	BioNLP11 ID-ggp	BioNLP11 ID-species
BC2GM	84.15 *↑*	**84.39** *↑*	84.01	83.45 *↓*
Ex-PTM	68.89 *↑*	67.51 *↑*	**68.80** *↑*	67.58 *↑*
NCBI-disease	84.90 *↓*	**85.44** *↓*	85.26 *↓*	85.24 *↓*
Linnaeus	78.46 *↑*	72.09 *↓*	73.21 *↓*	**76.88** *↓*

Bold: the best F1 score between sub-datasets. *↑*/ *↓*: positive / negative improvement comparing to STM

Ex-PTM dataset contains tags of protein category, and its best partner BioNLP11ID-ggp also contains that category of tags. Besides, as for Linnaeus and BioNLP11ID-species, these two datasets are the best pair and both contain tags of species category. It indicates that protein tags and species tags are the major factors for Ex-PTM and Linnaeus datasets, respectively, when BioNLP11ID as the auxiliary dataset. As for other tags, chemical and species tags in the BioNLP11ID dataset are hardly helpful to Ex-PTM dataset, while chemical and protein tags would make the performance of Linnaeus ever worse.

BC2GM and NCBI-disease datasets contain no tags of chemical, protein and species categories. In experiment results, we could observe that chemical and protein tags in BioNLP11ID dataset are helpful to BC2GM while species tags are harmful. For NCBI-disease dataset, all categories of tags make performance worse.

When a dataset contains multiple categories of tags, mutual influences may exist between them. For BC2GM datasets, chemical tags improve performance and species tags reduce performance, but the result of all tags is still positive. It indicates that categories of tags with the opposite effect would neutralize each other. For Ex-PTM dataset, all the categories of tags improve performance, and the performance of all tags is better than a single category of tags. Similarly, for NCBI-disease dataset, the merged result is worse. It demonstrates that the categories of tags with the same effect could cooperate and accumulate their effects. Exceptionally, for Linnaeus dataset, categories of tags are all negative, but the result of all tags is positive. We don’t have an analysis to explain this phenomenon if just base on the current experiment result, but it suggests that the influence of different categories of tags is not a simple linear calculation.

### Impact of dataset size

In this part, we discover the performance of CS-MTM on smaller datasets. Using reduced-size main datasets, we record the performance (precision, recall, F1) of different situations. The results of CS-MTM in this experiment are produced using the best pairs in “Performance with different auxiliary datasets” section. The reduced-size datasets are produced by removing sentences in training sets randomly, and the development sets and test sets are not modified. To compare with, we also use the reduced-size dataset on baseline single-task model. The results are shown in Table 6. The better F1 scores for each training set size are bold.

**Table 6:** Impact of dataset size

		Full-size STM	Full-size CS-MTM	50%-size STM	50%-size CS-MTM	25%-size STM	25%-size CS-MTM	10%-size STM	10%-size CS-MTM
BC2GM	Precision	84.00	83.12	82.37	79.37	77.82	79.44	73.19	72.95
	Recall	83.82	85.74	80.77	85.05	79.57	78.98	73.59	75.39
	F1	83.91	**84.41**	81.56	**82.12**	78.69	**79.21**	73.39	**74.15**
Ex-PTM	Precision	70.83	74.73	67.74	68.18	57.46	54.00	42.47	50.69
	Recall	64.12	69.56	58.62	67.48	53.69	63.97	50.27	41.68
	F1	67.31	**72.05**	62.85	**67.83**	55.51	**58.56**	**46.04**	45.75
NCBI-disease	Precision	88.45	86.59	84.03	84.72	81.52	81.00	81.02	79.32
	Recall	83.78	86.42	84.56	84.76	76.50	81.00	68.59	74.40
	F1	86.05	**86.50**	84.30	**84.74**	78.93	**81.00**	74.29	**76.78**
Linnaeus	Precision	92.86	89.81	91.77	88.92	89.90	90.20	90.80	85.98
	Recall	67.62	76.12	68.11	72.95	67.62	68.29	52.65	51.33
	F1	78.25	**82.40**	78.19	**80.15**	77.18	**77.73**	**66.65**	64.29

Bold: the better F1 scores between STM and CS-MTM for each dataset size

For STM and CS-MTM, the F1 score decreases when the size of training data is limited. When the training set is reduced and the test set is kept, the missing of information in removed sentences makes the model produce worse results. In CS-MTM, the missing information could be found in auxiliary datasets, so CS-MTM could improve the performance back if a suitable auxiliary dataset is chosen.

For 50%-size and 25%-size datasets, CS-MTM outperforms STM on F1 score by providing a higher recall score. But for 10%-size datasets, CS-MTM outperforms STM on BC2GM and NCBI-disease datasets and degrades on Ex-PTM and Linnaeus datasets. In this case, our CS-MTM may not learn missing information from auxiliary dataset well.

### Performance with different word embeddings

In this part, we discover the performance of STM and CS-MTM by using different pre-trained word embeddings. In our previous experiments, we just use the pre-trained GloVe to produce our word embeddings. Our CS-MTM model may have better performance when using other word embeddings. In this experiment, we obtain the performance with several different pre-trained Word2Vec and compare them with the performance with the original pre-trained GloVe. The results are shown in Table 7. The best F1 scores for the model on each dataset are bold.

**Table 7:** Performance with different word embeddings

	STM				CS-MTM			
	BC2GM	Ex-PTM	NCBI-disease	Linnaeus	BC2GM	Ex-PTM	NCBI-disease	Linnaeus
PMC	84.22	66.09	85.24	76.87	85.07	70.61	84.32	80.00
PubMed	84.15	66.86	85.21	71.23	83.84	70.66	84.99	74.63
PMC+PubMed	84.35	66.57	84.39	75.07	**85.18**	72.03	85.34	76.71
PMC+PubMed +Wikipedia	**84.71**	65.71	84.46	76.87	84.10	71.79	85.27	78.99
Our GloVe	83.91	**67.31**	**86.05**	**78.25**	84.41	**72.05**	**86.50**	**82.40**

Bold: the best F1 scores for the model on each dataset

Four pre-trained Word2Vec word embeddings are used in this experiment. One trains with PMC corpus, one trains with PubMed corpus, one trains with PMC + PubMed corpora, one trains with PMC + PubMed + Wikipedia corpora. These pre-trained Word2Vec word embeddings are available at http://bio.nlplab.org/. They report that Word2Vec was run using the skip-gram model with a window size of 5, hierarchical softmax training, and a frequent word subsampling threshold of 0.001 to create 200-dimensional vectors.

For STM, we have the best performance on BC2GM dataset when choosing PMC + PubMed + Wikipedia word embedding, and the best performance on the other three datasets is achieved by our original GloVe word embedding. For CS-MTM, PMC + PubMed on BC2GM and other three datasets on GloVe word embedding can produce the best performance. This phenomenon shows that different word embeddings can produce discrepant performance.

Our GloVe word embedding achieves good performance on three datasets, but the coverage of Glove might be relatively small because it is not trained with the biomedical corpus. An important reason is that CNN in the embedding layer builds character embeddings to compensate for the missing of words. Besides, according to the overall performance, GloVe embeddings work better with our models than Word2Vec embeddings. But on certain datasets, such as BC2GM, character embeddings may not work well, and using word embedding which trains with specialized corpus can improve the performance.

### Case study

In this part, we use some examples from datasets to illustrate the effect of the multi-task model. The examples are shown in Table 8.

**Table 8:** Case Study: Bold text: ground-truth entity; Underlined text: model prediction

Main dataset: Ex-PTM Auxiliary dataset: BioNLP09		
Case 1	STM	The myristoylation of **Nef** and its membrane localization were essential for these effects.
	CS-MTM	The myristoylation of **Nef** and its membrane localization were essential for these effects.
	Auxiliary data	Human immunodeficiency virus type 1 **Nef** protein inhibits NF-kappa B induction in human T cells.
Description	The training data of auxiliary dataset directly provides entity information about Nef protein.	
Main dataset: Ex-PTM Auxiliary dataset: BioNLP09		
Case 2	STM	Vitamin K deficiency is a relatively common condition in neonates.
	CS-MTM	Vitamin K deficiency is a relatively common condition in neonates.
	Auxiliary data	Ascorbic acid (ascorbate or vitamin C) has been shown to suppress the induction of HIV in...
		In conclusion, we demonstrate that the vitamin E derivative TCP succinate prevents monocytic...
Description	The training data of auxiliary dataset indirectly provides information that Vitamin is not protein.	
Main dataset: Linnaeus Auxiliary dataset: BC5CDR		
Case 3	STM	He slept well at night, ate more than his mother thought was good for him, and was able to...
	CS-MTM	He slept well at night, ate more than his mother thought was good for him, and was able to...
	Auxiliary data	During the night **clomipramine** ingestion altered the complete sleep architecture in that it suppressed REM sleep and the sleep cycles and induced increased wakefulness.
Description	The training data of auxiliary dataset directly provides information that sleep don’t belong to species.	

Case 1 and 2 are picked from the test set of Ex-PTM. The main dataset, Ex-PTM, and the auxiliary dataset, BioNLP09, only have entity tags of protein category. In case 1, STM cannot recognize the entity Nef but CS-MTM can find it out, because the training data of auxiliary dataset directly provides entity information about Nef protein. In case 2, STM recognizes Vitamin K as a protein entity, which is incorrect. For the CS-MTM, in the training data of auxiliary dataset, there is no information about Vitamin K, but other Vitamins, such as Vitamin C and Vitamin E, appear in the dataset. The character embedding in the model can capture the morphological information; therefore, the multi-task model can recognize these Vitamins as non-protein entities.

Case 3 is picked from the test set of Linnaeus. Linnaeus contains entity tags of species category, but the auxiliary dataset, BC5CDR, have no species entity tags. In case 3, STM recognizes *slept* as a species entity. Because our model use no pre-defined feature, such as Part-of-Speech feature, STM may not learn that *slept* is not an entity if there are few appearances of this word. For the CS-MTM, it can learn from auxiliary training data which exists the information of *sleep*; therefore, CS-MTM can recognize it as a non-species entity.

## Discussion

In this part, we compare our models with other BioNER models as well as the state-of-the-art models.

For the multi-task model from Crichton et al. [23], they experiment with many BioNER datasets. They report their best model achieves the F1 of 73.17% on BC2GM, 74.90% on Ex-PTM, 80.37% on NCBI-disease, and 84.04% on Linnaeus. Our model has better performance on BC2GM and NCBI-disease datasets, because both word embedding and character embedding are used as input in our model, while only word embedding is used in their model. In Crichton’s work, many more combinations of datasets are tried in the experiment, so this could be the reason why they have better performance on Ex-PTM and Linnaeus.

For the multi-task model from Wang et al. [19], they achieve the F1 of 83.14% on BC2GM and 86.37% on NCBI-disease. Our model outperforms their model on these two datasets, because we use shared and private Bi-LSTMs to capture different features, as well as the gated interaction unit to make use of features from the auxiliary dataset.

For the BioBERT model from Lee et al. [30], they report their best model achieves the F1 of 84.40% on BC2GM, 89.36% on NCBI-disease, and 89.81% on Linnaeus. Their model outperforms ours because BioBERT has much more trainable parameters than ours. In BioBERT’s paper, the authors don’t report the number of parameters, but BioBERT should be similar to the original BERT which has more than 100M parameters to train.

For the CollaboNet model from Yoon et al. [31], they achieve the F1 of 78.56% on BC2GM and 86.36% on NCBI-disease. This model uses a special structure to achieve good performance, but our model uses multi-task learning to achieve better performance on BC2GM dataset.

As for state-of-the-art models, BioCreative II Gene Mention Tagging System [10] achieves the F1 of 87.21% on BC2GM dataset, MO-MTM from Crichton et al. [23] achieves the F1 of 74.90% on Ex-PTM dataset, BioBERT [30] achieves the F1 of 89.36% on NCBI-disease dataset, and the original LINNAEUS system [32] achieves the F1 of 95.68% on Linnaeus dataset. Although BioCreative II and LINNAEUS system have the best performance on certain datasets, they rely heavily on hand-craft features which are not used in our model. Besides, these systems can pre-process the input data or have some special process using field knowledge, which benefits the performance.

## Conclusion

In this paper, we propose a new multi-task learning framework for BioNER. We also implement some other multi-task models and compare our new model with them. Our proposed model achieves better performance, even if the size of the training data is smaller. Detailed analysis about best partners of datasets and influence between entity categories can provide guidance of choosing proper dataset pairs for multi-task training. Furthermore, our analysis suggests that the cross-sharing structure in our model is a key point to improve performance in the way of cross-dataset feature sharing.

Limitations to the work include that it is difficult to predict whether one dataset can help another before running the model. Another limitation is that the current implementation of the model may not produce promising results for all datasets, in our experiment we find the performance of the proposed model on Linnaeus dataset worse than the ADV-MTM.

There are several further directions with our cross-sharing multi-task model. First, training more datasets at the same time could provide more cross-dataset information and obtain better performance. Besides, we can adjust our cross-sharing structure to improve the performance on certain datasets or combine the current multi-task model with the newly proposed structure, such as BioBERT. Finally, our work may have entity type conflict problem, we could use an entity type unifier to recognize by source datasets in order to get the performance improvement.

## Data Availability

BioNER datasets are available at https://github.com/cambridgeltl/MTL-Bioinformatics-2016. Our implement of cross-sharing multi-task model is available at https://github.com/JogleLew/bioner-cross-sharing.

## Abbreviations

ADV-MTM: Adversarial multi-task model
Bi-LSTM: Bi-directional long short-term memory
BiLSTM-CRF: Bi-directional long short-term memory with conditional random field
BioNER: Biomedical named entity recognition
CRF: Conditional random field
CS-MTM: Multi-task model with cross-sharing structure
FS-MTM: Fully-shared multi-task model
LSTM: Long short-term memory
MTL: Multi-task learning
RNN: Recurrent neural network
SP-MTM: Shared-private multi-task model
STM: Single-task model
